# Transplantation of AQP1-overexpressing mitochondria attenuates periodontitis-associated bone loss by regulating mitochondria-ER contacts of macrophages

**DOI:** 10.1186/s12951-026-04084-z

**Published:** 2026-05-28

**Authors:** Shuqing Yang, Ming Zhang, Junbin Wei, Le Yu, Houze Li, Shuxuan Rong, Lingling Chen, Tingting Zhao, Junkun Zhan, Xiaoxiao Wang, Yunyi Xie, Yan Wang

**Affiliations:** 1 Hospital of Stomatology, Guanghua School of Stomatology, Guangdong Provincial Key Laboratory of Stomatology, Sun Yat-sen University, Guangzhou, 510055 China; 2 Department of Orthodontics, Wuwei People’s Hospital, Wuwei, 733000 China; 3 Oral Medical Center, Shenzhen Qianhai Taikang Hospital, No. 3099, Menghai Avenue, Nanshan District, Shenzhen, PR China; 4 Shenzhen Stomatology Hospital, 70 Guiyuan North Road, Luohu district, Shenzhen, PR China

**Keywords:** Periodontitis, Aquaporin 1, Engineered mitochondria, Immunometabolism, Osteoclast differentiation, Bone resorption

## Abstract

**Background:**

Periodontitis is a chronic inflammatory disease characterized by oxidative stress, immune dysregulation, and progressive alveolar bone loss. Conventional treatments such as mechanical debridement and antimicrobial therapy often fail to reverse bone destruction or restore periodontal homeostasis, underscoring the need for novel therapeutic strategies. Mitochondria have emerged as critical regulators of immune–metabolic signaling, and their genetic modification offers new opportunities for targeted intervention.

**Results:**

This study explored mitochondria engineered to overexpress aquaporin 1 (ovAQP1-mito) as a therapeutic approach for periodontitis. In vitro, ovAQP1-mito modulated macrophage activity and inhibited receptor activator of nuclear factor κB ligand (RANKL)-induced osteoclast differentiation, suggesting direct immunometabolic effects. In vivo, transplantation of ovAQP1-mito into a ligature-induced mouse model attenuated alveolar bone loss, reduced inflammatory cell infiltration, and preserved periodontal architecture. Mechanistic studies further demonstrated that ovAQP1-mito enhanced SigmaR1 expression, facilitated endoplasmic reticulum–mitochondria communication, and suppressed osteoclastogenic signaling.

**Conclusion:**

These findings indicate that ovAQP1-mito exerts dual regulatory effects by mitigating inflammation and bone resorption through ER-mito contacts and immune–metabolic modulation. Such mitochondria-based engineering may represent a promising therapeutic strategy for periodontitis and potentially other inflammatory bone disorders.

**Graphical abstract:**

**Figure:**
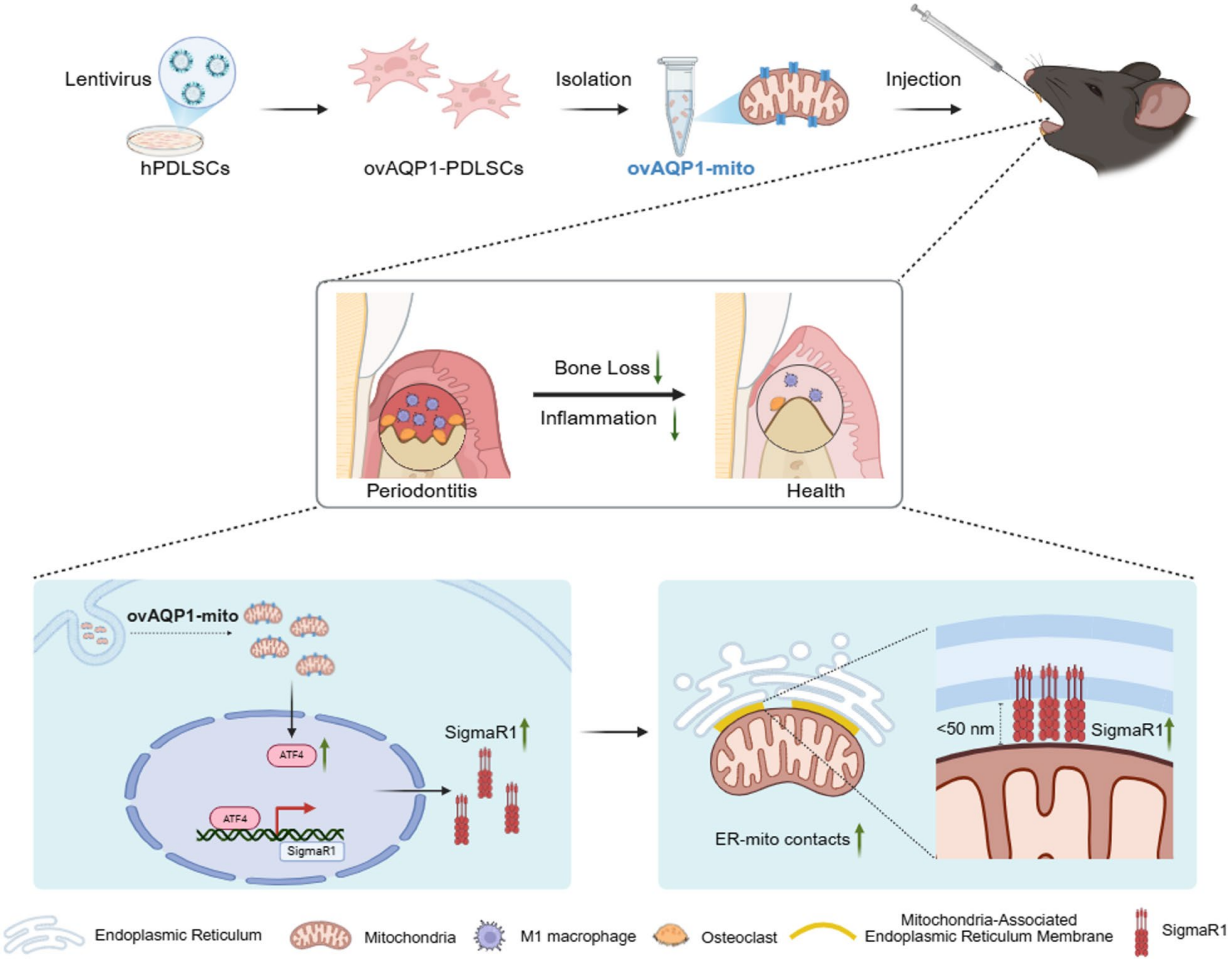

**Supplementary Information:**

The online version contains supplementary material available at 10.1186/s12951-026-04084-z.

## Background

Periodontitis is the most common osteolytic inflammatory disease, ranking sixth among the most prevalent conditions worldwide [1], affecting nearly 50% of the global population, and being a primary cause of adult tooth loss [2–4]. Current treatments often fail to completely regenerate the alveolar bone, hence necessitating novel therapeutic approaches. Periodontitis is an oxidative stress-related disease, and its onset and progression are closely associated with mitochondrial function [5–7]. Osteoclasts, which are derived from bone marrow monocyte/macrophage lineages, are the sole executors of bone resorption [8]. Inflammatory conditions promote their differentiation from circulating or resident macrophages, with myeloid progenitors giving rise to either bone macrophages or osteoclasts, depending on environmental cues [9]. Bone loss in periodontitis is primarily mediated by osteoclasts. Inflammation elevates ROS and nuclear factor kappa-B ligand (RANKL) levels, leading to mitochondrial dysfunction, disrupting bone homeostasis, and overactivating osteoclasts [10–12]. Thus, osteoclasts are the key drivers of periodontal destruction and, at the same time, are potential therapeutic targets.

Mitochondrial dysfunction is a key feature in the pathology of periodontitis. Gingival tissues from patients show decreased mtDNA expression and other mitochondrial abnormalities [13]. Pro-inflammatory cytokines and ROS damage mitochondrial function in stem cells, hindering bone regeneration and immune regulation [14–16]. Moreover, chronic inflammation activates glycolysis in osteoclasts by impairing mitochondrial activity, leading to excessive lactate production and bone resorption [17]. Therefore, mitochondria are the central regulators of periodontal tissue homeostasis.

Mitochondrial transplantation, which delivers functional mitochondria from healthy cells to impaired cells, has emerged as a potential therapeutic strategy. Mesenchymal stromal cells (MSCs) are ideal donors owing to their high mitochondrial content and bioenergetic capacity. MSCs-derived mitochondria have been shown to repair injured cells in various diseases [18, 19]. Advances in mitochondrial proteomic profiling and gene editing have enabled us to target nuclear genes encoding mitochondrial proteins [20], providing powerful tools and new perspectives to understand their role in disease pathogenesis better.

Aquaporin 1(AQP1) is a classical water channel protein that mediates osmotically driven water transport and has also been implicated in cell migration, angiogenesis, and inflammatory outcomes [21–23]. At the organelle level, aquaporins such as AQP8 and AQP9 localize to the mitochondrial inner membrane [24–26], while evidence from retinal hyperosmotic stress and endothelial AQP1 deficiency links AQP1 to mitochondrial swelling, cristae disruption, and altered oxygen transport [27–29], suggesting a potential role in metabolic regulation. Reports in macrophages further indicate that AQP1 participates in polarization and antioxidant responses [30–32], providing a rationale for its immunomodulatory relevance.

In this study, we demonstrated that inflammation impairs the mitochondrial transfer from PDLSCs to macrophages. Moreover, mitochondria derived from PDLSCs of periodontitis patients (I-mito) exhibited reduced capacity to suppress osteoclast differentiation and to alleviate oxidative stress in inflammatory macrophages compared with mitochondria from healthy donors(H-mito), potentially attributable to differences in AQP1 abundance. Engineered AQP1-overexpressing mitochondria (ovAQP1-mito) inhibited osteoclast differentiation and ameliorated bone loss in a mouse periodontitis model. Mechanistically, ovAQP1-mito upregulated SigmaR1 in macrophages, promoting the restoration of mitochondria–endoplasmic reticulum contacts (MERCs). The enhanced SigmaR1 expression was functionally associated with reduced osteoclastogenic activity.

## Methods

### Animals

All experiments were performed on C57BL/6 male mice aged 6–8 weeks. They were housed in the Animal Facility at Sun Yat-sen University under a 12 h light/dark cycle. Water and chow were provided ad libitum. All experimental procedures were performed in compliance with the institutionally approved animal research protocol (Sun Yat-sen University; SYSU-IACUC-2024–002082), guaranteeing adherence to the ethical guidelines and regulatory standards.

### Ligature-induced periodontitis

In the experimental group, 5 − 0 silk ligatures were tied around the maxillary second molars on one side of the mice (*n* = 30), whereas the control mice (*n* = 6) remained unligated. The ligatures were checked every 3 days. After 2 weeks, the ligatures were removed, and the experimental mice (*n* = 30) were randomly divided into five groups. The periodontitis group received no intervention, whereas the mitochondrial group was administered 5 µL of mitochondrial solution to both the buccal and palatal aspects of the periodontal ligament of the maxillary second molar on one side, resulting in a total injection volume of 10 µL containing 25 µg of mitochondria [33]. The PBS group received an equal volume of PBS. The injections were administered twice a week, and periodontal tissues were collected after 3 weeks [34].

### Cell culture of THP-1 macrophages

THP-1 macrophages (Procell, China) were cultured in RPMI 1640 (Gibco, USA) supplemented with 10% FBS (NEWZERUM, New Zealand), 100 U/mL streptomycin/penicillin (Gibco, USA), 20 mM HEPES (Procell, China), and 20 nM 2-mercaptoethanol (Gibco, USA).

### Isolation and culture of human PDLSCs

Premolars from healthy donors(18–25 years old) undergoing orthodontic treatment without systemic diseases or recent medications, as well as from patients with periodontitis were collected. PDLSCs from healthy donors were termed as H-PDLSCs and those from patients with periodontitis were termed as I-PDLSCs. Extracted caries-free teeth with intact crowns were transported to the laboratory within 2 h. The teeth were held by forceps at the cervical region and rinsed 3–4 times in α-MEM (Gibco, USA). Thereafter, they were immersed in α-MEM containing 10% FBS and 100 U/mL streptomycin/penicillin. Periodontal tissue from the middle section of the root was scraped with a sterile blade, collected into a 15 mL tube, and centrifuged at 1000 rpm for 5 min. The pellet was digested with type I collagenase at 37 °C for 1 h and vortexed every 5 min. After digestion, the cells were centrifuged, resuspended, and seeded in T25 flasks (Biofil, China). Complete medium (α-MEM +10% FBS +100 U/mL streptomycin/penicillin) was changed every 3 days, and cells were sub-cultured or cryopreserved accordingly. The study protocol of PDLSCs isolation was approved by the Medical Ethics Committee of the Hospital of Stomatology at Sun Yat-sen University (KQEC-2022-116−01).

### Extraction and culture of primary bone marrow-derived macrophages (BMDMs)

BMDMs were isolated from mouse femurs and tibias using a well-established protocol [35]. Harvested cells were seeded and differentiated in DMEM (Gibco, USA) supplemented with 10% FBS, 25 ng/mL recombinant mouse macrophage colony-stimulating factor (M-CSF; Peprotech, USA), and 100 U/mL streptomycin/penicillin. The medium was changed every 3 days.

### Intercellular mitochondrial transfer assay

BMDMs were isolated and seeded in 24-well plates at 2.4 × 10^5^ cells per well and differentiated with 25 ng/mL M-CSF. THP-1 macrophages were plated at the same density in separate plates. H-PDLSCs were cultured in 10-cm dishes. After 36 h, 2 µg/mL Lipopolysaccharide derived from *Porphyromonas gingivalis* (*P. gingivalis* LPS, hereafter referred to as LPS) (Invitrogen, USA) was added to H-PDLSCs and macrophages in the designated groups. After 12 h, PDLSCs were stained with 100 nM MitoTracker Deep Red FM (Thermo Fisher Scientific, USA) for 20 min in basal medium. The cells were then harvested, centrifuged at 1000 rpm for 5 min, resuspended in α-MEM, and counted. The labeled PDLSCs were co-cultured with BMDMs or THP-1 macrophages at 1:1 ratio. After 12 h, the actin cytoskeleton was labeled with 100 nM Actin-Tracker Green (Beyotime Biotechnology, China) for 60 min, and the nuclei were stained with 1 µg/mL DAPI (Abcam, USA) for 8 min. Fluorescence images were captured using a Zeiss LSM 980 confocal microscope (Zeiss, Germany) to assess mitochondrial transfer.

### Isolation of mitochondria

Mitochondria were isolated from PDLSCs using a kit (Thermo Fisher Scientific, USA) according to the manufacturer’s instructions. The cells were harvested, centrifuged, resuspended, and transferred to 2 mL microcentrifuge tubes. Next, 800 µL Reagent A was added, vortexed at medium speed for 5 s, and incubated on ice for 2 min. After adding 10 µL Reagent B, the samples were vortexed at the maximum speed for 5 s, and kept on ice for 5 min with brief vortexing every minute. Thereafter, 800 µL Reagent C was added and mixed gently. The lysate was centrifuged at 700 × g for 10 min. The supernatant was collected and centrifuged at 12,000 × g for 15 min. The pellet was washed with 500 µL Reagent C and centrifuged again at 12,000 × g for 5 min. The final mitochondrial pellet was resuspended and stored on ice. All centrifugations were performed at 4 °C. It should be emphasized that both in vivo injections and in vitro applications were completed within 2 h [36, 37] after mitochondrial isolation.

### Detection of mitochondrial uptake

BMDMs and THP-1 macrophages were seeded in 24-well plates, whereas H-PDLSCs were cultured in 10-cm dishes. After 36 h, 2 µg/mL LPS was added to the H-PDLSCs and macrophages in the designated groups. After another 12 h, mitochondria were isolated from PDLSCs, labeled with 100 nM MitoTracker Deep Red FM for 20 min, and added to macrophages at a donor-to-recipient ratio of 3:1 [38]. After another 12 h, macrophage actin cytoskeleton was stained with 100 nM Actin-Tracker Green for 60 min, and the nuclei were stained with 1 µg/mL DAPI for 8 min. Mitochondrial uptake was visualized by Zeiss LSM 980 confocal microscopy. Quantitative analysis was performed by flow cytometry using FACSDiva (BD Biosciences, USA) and FlowJo 10.8.1 software.

### Staining of osteoclasts using tartrate-resistant acid phosphatase (TRAP)

BMDMs were seeded at a density of 1 × 10⁵ cells per well in 48-well plates. Mitochondria isolated from the respective PDLSC groups were added at a donor-to-recipient ratio of 3:1, together with DMEM supplemented with 25 ng/mL M-CSF and 40 ng/mL RANKL (Amizona, China). Cells were cultured for 7 days to induce osteoclast differentiation. For THP-1–derived macrophages, a comparable induction protocol was employed, except that the differentiation period was extended to 14 days.

Following induction, cells were subjected to tartrate-resistant acid phosphatase (TRAP) staining using a commercial kit (Servicebio, China) according to the manufacturer’s instructions, and images were captured using a light microscope (Zeiss, Germany).

To modulate SigmaR1 signaling during osteoclast differentiation, cells were pretreated with the SigmaR1 agonist PRE-084 (MCE, China, 10 µM) or the antagonist NE-100 (MCE, China, 1 µM) for 30 min prior to RANKL stimulation. ATF4 activity was inhibited by treatment with ATF4-IN-2 (MCE, China, 400 nM) for 6 h or by transfection with ATF4-specific siRNA (Sangon Biotech, China, 60 nM) for 48 h before the initiation of osteoclast differentiation. All pharmacological treatments and genetic interventions were maintained as indicated throughout the differentiation period.

### F-actin staining

Coverslips were placed in 48-well plates, and BMDMs were seeded at 1 × 10⁵ cells/well. According to the experimental design, mitochondria were added at a donor-to-recipient ratio of 3:1 in DMEM supplemented with 25 ng/mL M-CSF and 40 ng/mL RANKL. For THP‑1 macrophages, an analogous induction protocol was applied, except that the induction period was extended to 14 days. After 7 days of induction (14 days for THP-1 macrophages), F-actin was stained with 100 nM TRITC-phalloidin (Yeason, China), and the nuclei were counterstained using DAPI-containing mounting medium (Abcam, USA). Fluorescent images were captured using a Zeiss LSM 980 confocal microscope.

### Intracellular ROS detection

BMDMs and THP-1 macrophages were seeded at 2.4 × 10⁵ cells/well in 24-well plates. LPS (2 µg/mL) was added for 24 h to macrophages in the experimental groups. Mitochondria isolated from PDLSCs were added to complete DMEM at a donor-to-recipient ratio of 3:1 and incubated for 24 h. Intracellular ROS levels were assessed using a DCFH-DA-based ROS assay kit (Dojindo Laboratories, Japan). After washing twice with PBS, the cells were incubated with 10 µM DCFH-DA at 37 °C in the dark for 20 min, followed by three washes with PBS. Fluorescence was visualized using a Zeiss LSM 980 confocal microscope.

### Construction of AQP1-overexpressing PDLSCs (ovAQP1-PDLSCs) and isolation of ovAQP1-mito

A lentiviral system was used to generate H-PDLSCs stably overexpressing AQP1. Specific primers were designed based on the GenBank sequence (NM_198098.4), and AQP1 was amplified and inserted into the pMSCVpuro vector to construct pMSCV-AQP1, which was verified by sequencing. The recombinant and lentiviral packaging plasmids were co-transfected into 293 T cells. After virus purification, concentration, and titer determination, H-PDLSCs were infected and selected with puromycin. Stable AQP1-overexpressing H-PDLSCs (ovAQP1-PDLSCs) and vector control (ovCTR-PDLSCs) were used in this study. The corresponding mitochondria, isolated following the method mentioned above, are referred to as ovAQP1‑mito and ovCTR‑mito, respectively.

### Western blotting

Cells or mitochondria were lysed in RIPA buffer (Beyotime Biotechnology, China) with 100× Protease Inhibitor Cocktail (CoWin Biotech, China), and protein concentrations were measured using the BCA assay kit (Thermo Fisher Scientific, USA). Samples were then mixed with 5× SDS-PAGE loading buffer (CoWin Biotech, China), boiled at 95 °C for 10 min, separated on 10% SDS-PAGE at 80 V, and transferred to PVDF membranes (Millipore, USA) at 200 mA. Membranes were blocked with 5% skimmed milk (Beyotime Biotechnology, China) for 1 h and incubated overnight at 4 °C with the following primary antibodies: anti-AQP1 (Abcam, USA), anti-TOB2 (Abcam, USA), anti-A2M (Cell Signaling Technology, USA), anti-COX IV (Abcam, USA), anti-SigmaR1 (Cell Signaling Technology, USA), and anti-β-actin (Proteintech, China). After TBST washing, HRP-conjugated secondary antibodies (Proteintech, China) were applied for 1 h at room temperature. Signals were detected using ECL (Beyotime Biotechnology, China) and imaged with a ChemiDoc^™^ MP system (Bio-Rad, USA). Band intensities were quantified using ImageJ 1.54p (NIH, USA). Cellular proteins were normalized to β-actin and mitochondrial proteins to COX IV.

### RT-qPCR

 Total RNA was extracted from PDLSCs, BMDMs, and THP-1 cells using an Ultrapure RNA Kit (CoWin Biotech, China) according to the manufacturer’s instructions. Genomic DNA was removed by treatment with 4× gDNA wiper mix (Vazyme, China). Subsequently, 1 µg of RNA was reverse-transcribed using 5× HiScript III RT SuperMix (Vazyme, China). Quantitative PCR was conducted using 2× RealStar Fast SYBR qPCR Mix (Genstar, China) on a LightCycler 96 system (Roche, Switzerland). Primer sequences for the target genes are listed in Table S1. Data were analyzed using the ∆∆Ct method.

### Immunofluorescence-based evaluation of macrophage polarization

Coverslips were placed in 24-well plates, and BMDMs were seeded at 2 × 10^5^ cells per well. After treatments according to the experimental groups, the cells were fixed with 4% paraformaldehyde, permeabilized with 0.1% Triton X-100, and blocked with 5% BSA. Primary antibodies against iNOS (Cell Signaling Technology, USA) or Arg1 (Proteintech, China) were applied, followed by overnight incubation at 4 °C. The cells were then washed thrice with PBS and incubated with Alexa Fluor 488- or Alexa Fluor 555-labeled secondary antibodies (Abcam, USA) for 1 h at room temperature. After three washes with PBS, the cells were mounted with a DAPI-containing medium, sealed, and fluorescence images were captured using a Zeiss LSM 980 microscope.

### Flow cytometric assessment of macrophage polarization

BMDMs and THP-1 macrophages were processed according to the experimental groups and maintained on ice. Cells were washed twice with cold PBS, scraped gently, and transferred to flow cytometry tubes. After centrifugation at 1300 rpm for 5 min at 4 °C, cells were resuspended in PBS and stained with the PE-conjugated anti-CD86 antibody and BV421-conjugated anti-mouse CD206 antibody (Biolegend, USA). Data were acquired using FACSDiva and analyzed using the FlowJo 10.8.1 software.

### Micro-CT Imaging

Micro-CT imaging (µCT50; SCANCO, Switzerland) was performed immediately after periodontal intervention. Three-dimensional reconstructions and representative sectioned micro-CT images were generated using the Mimics software (Mimics 17.0; Materialize, Leuven, Belgium). Alveolar bone loss was evaluated by measuring the vertical distance from cemento–enamel junction (CEJ) to alveolar bone crest (ABC) at six anatomical locations, including three points on the palatal side (mesial, middle, and distal) and three points on the buccal side (mesial, middle, and distal).

### Histological analysis

Mouse maxillary specimens were fixed in 4% paraformaldehyde for 48 h and decalcified in 10% EDTA for 3 weeks. Samples were embedded in paraffin and sectioned at 4 μm for hematoxylin & eosin (H&E) and Masson’s trichrome staining to assess histological changes. Osteoclasts were detected by TRAP staining using a TRAP staining kit (Njjcbio, China). Immunohistochemistry was used to evaluate bone mineralization. Sections were deparaffinized, rehydrated, rinsed in PBS, and subjected to heat-mediated antigen retrieval in citrate buffer (Dingguo, China). After blocking, the sections were incubated overnight at 4 °C with anti-bone sialoprotein (BSP) primary antibody (Zenbioscience, China). The following day, the sections were incubated with an anti-rabbit secondary antibody and developed using DAB (Servicebio, China). The slides were scanned using a Leica digital slide scanner (Leica, Germany).

### Confocal imaging of MERCs

The BMDMs were seeded in 96-well plates at a density of 4 × 10⁴ cells per well. After 24 h, mitochondria isolated from the indicated PDLSC groups were added at a donor-to-recipient ratio of 3:1 in DMEM supplemented with 25 ng/mL M-CSF and 40 ng/mL RANKL (Amizona, China). Control wells received medium containing M-CSF alone. After 3 days, the medium was refreshed with the same concentrations of mitochondria, M-CSF, and RANKL. Three days later, BMDM mitochondria were labeled with 100 nM MitoTracker Deep Red FM (Thermo Fisher Scientific, USA) for 20 min. ER visualization was performed using established approaches optimized for the corresponding imaging conditions. Specifically, ER was labeled with 1 µM ER-Tracker Green (Thermo Fisher Scientific, USA) for 30 min. Alternatively, ER morphology was assessed by immunofluorescence staining of the ER-resident protein calnexin. For calnexin immunostaining, cells were washed twice with PBS, fixed with 4% paraformaldehyde for 15 min at room temperature, washed twice with PBS, permeabilized with 0.1% Triton X-100 (v/v in 1× PBS) for 10 min, and blocked with 5% BSA (w/v in 1× PBS) for 1 h at room temperature. Cells were then incubated with an anti-calnexin primary antibody (Abcam, USA;1:200, diluted in 5% BSA/PBS) overnight at 4 °C, followed by three PBS washes and incubation with a 488-conjugated anti-rabbit secondary antibody (1:200, diluted in 5% BSA/PBS) for 1 h at room temperature in the dark. After three additional washes with PBS, nuclei were counterstained using a DAPI-containing mounting medium, and samples were mounted for imaging. Imaging was performed using structured illumination microscopy (SIM; NanoInsights, China), and fluorescence data were analyzed using FlowJo 10.8.1 software.

### Transmission electron microscopy (TEM)

For ultrastructural analysis, isolated mitochondrial pellets or BMDM monolayers were fixed in commercial fixative (Servicebio, China) following the manufacturer’s instructions. Samples were then washed with phosphate buffer (PBS) and post-fixed with osmium tetroxide. After sequential dehydration in ethanol, specimens were infiltrated with Epon-Araldite resin and polymerized in molds. Ultrathin sections were prepared and contrasted with uranyl acetate followed by lead citrate. Images were acquired using an HT7800 transmission electron microscope (Hitachi, Japan).

To evaluate MERCs, the mitochondrial outer membrane and ER membrane were delineated. ER membrane segments located < 50 nm from the mitochondrial outer membrane were considered as Mitochondria-Associated Endoplasmic Reticulum Membranes(MAMs). The MAMs coverage ratio for each mitochondrion was calculated according to: MAM Ratio = MAM length (nm)/Mitochondrial perimeter (nm).

#### Statistical analysis

All experiments were performed in triplicate. Statistical analyses were conducted using GraphPad Prism version 10.0, and the data are presented as means ± standard deviation. Comparisons between two groups were assessed using independent two-tailed Student’s *t-tests*, and comparisons across more than two groups were evaluated using one-way ANOVA. Statistical significance was defined as *P* < 0.05, with thresholds denoted as *P < 0.05, **P < 0.01, *** *P* < 0.001. Spearman’s correlation analysis was used to assess the relationships across the parameters described in the results.

## Results

### Inflammation impairs mitochondrial transfer from PDLSCs to macrophages

MSCs maintain skeletal homeostasis by coupling bone formation and resorption through communication with macrophages, among which mitochondrial transfer represents a key mode of intercellular interaction. To explore whether periodontitis affects this process, we examined intercellular mitochondrial transfer from PDLSCs to key periodontal immune cells, including BMDMs, neutrophils, CD4^+^ T cells, and CD19^+^ B cells. Mitochondria purified from PDLSCs were fluorescently labeled and co-cultured with the immune cells. Notably, mitochondrial uptake was observed in neutrophils and macrophages, consistent with previous findings showing that mitochondria from osteogenic lineage cells can be transferred to myeloid cells [39] (Figure S1).

**Fig. 1:**
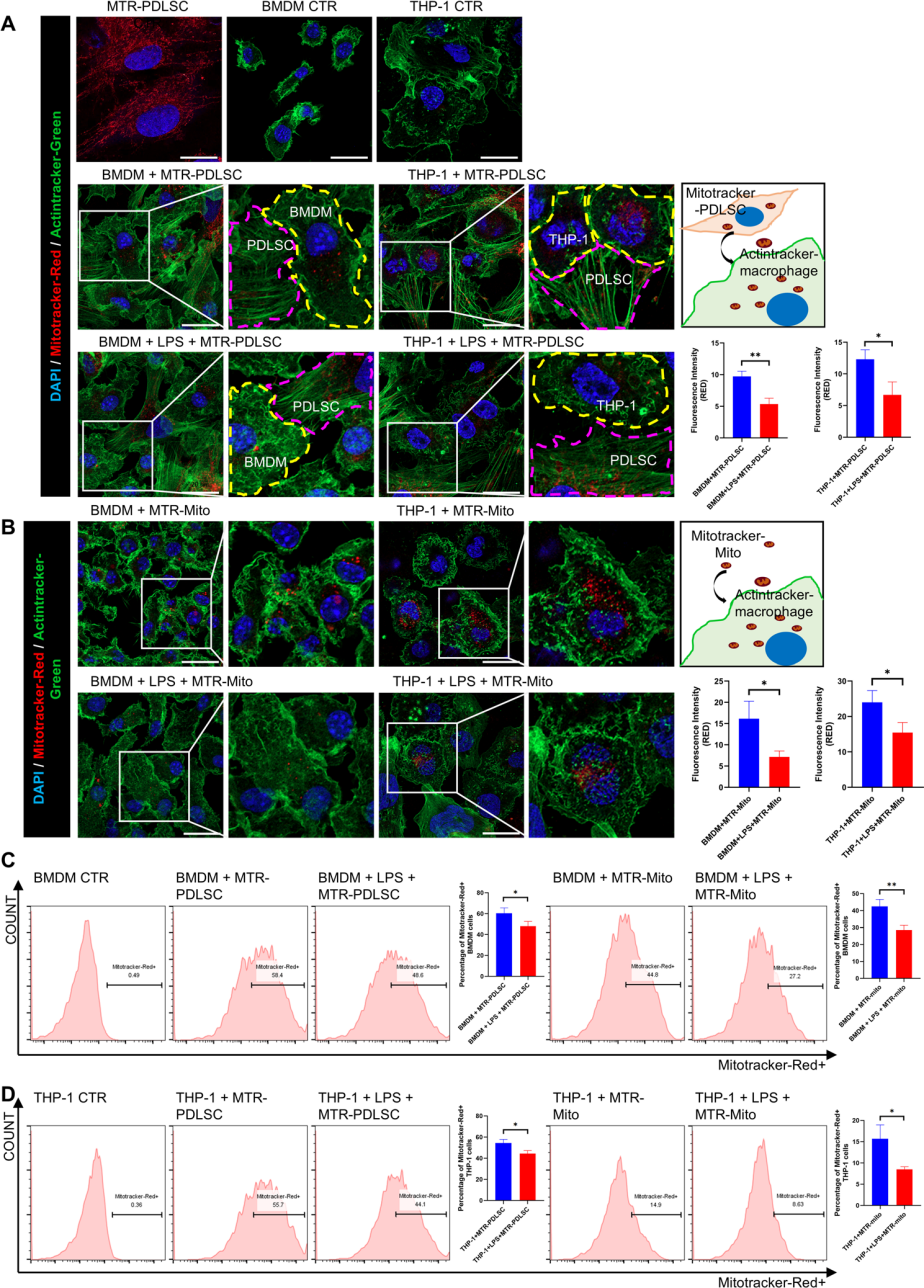
Inflammation impairs mitochondrial transfer from PDLSCs to macrophages. **A** Confocal images showing mitochondrial transfer from PDLSCs to BMDMs or THP-1 cells under normal conditions or in the presence of LPS (left, scale bar: 20 μm). Purple dashed outlines indicate PDLSCs, and yellow dashed outlines indicate BMDMs/THP-1 cells. top right: schematic diagram; bottom right: statistical analysis of red fluorescence intensity in BMDMs/THP-1 cells. **B** Confocal images showing uptake of exogenous mitochondria by BMDMs or THP-1 cells under normal conditions or in the presence of LPS (left, scale bar: 20 μm). Top right: schematic diagram; bottom right: statistical analysis of red fluorescence intensity in BMDMs/THP-1 cells. **C** Flow cytometry (left) and quantitative analysis (right) of the proportions of MitoTracker Red-positive cells in BMDMs. **D** Flow cytometry (left) and quantitative analysis (right) of the proportions of MitoTracker Red-positive cells in THP-1 cells. MTR-PDLSCs indicates PDLSCs labeled with MitoTracker Deep Red. MTR-Mito indicates mitochondria labeled with MitoTracker Deep Red. *P < 0.05, **P < 0.01

Given the altered inflammatory microenvironment in periodontitis, we investigated whether inflammatory stimulation impairs mitochondrial transfer. To simulate periodontitis-related inflammation, macrophages, including murine BMDMs and PMA-induced human THP-1 macrophages (Figure S1), were treated with LPS. Under both direct contact and non-contact co-culture conditions, we observed efficient mitochondrial transfer to healthy macrophages, whereas transfer efficiency was remarkably reduced under inflammatory conditions (Fig. 1A, B).

Flow cytometry was used to quantify these differences. Consistent with the fluorescence microscopy results, inflammation significantly suppressed the mitochondrial uptake by both BMDMs and THP-1 macrophages (Fig. 1C, D). The results suggested that inflammatory conditions impair PDLSCs-to-macrophage mitochondrial transfer, potentially disrupting cellular communication and immune homeostasis.

## H-mito inhibits macrophage osteoclastogenesis and reduce ROS levels more potently

**Fig. 2:**
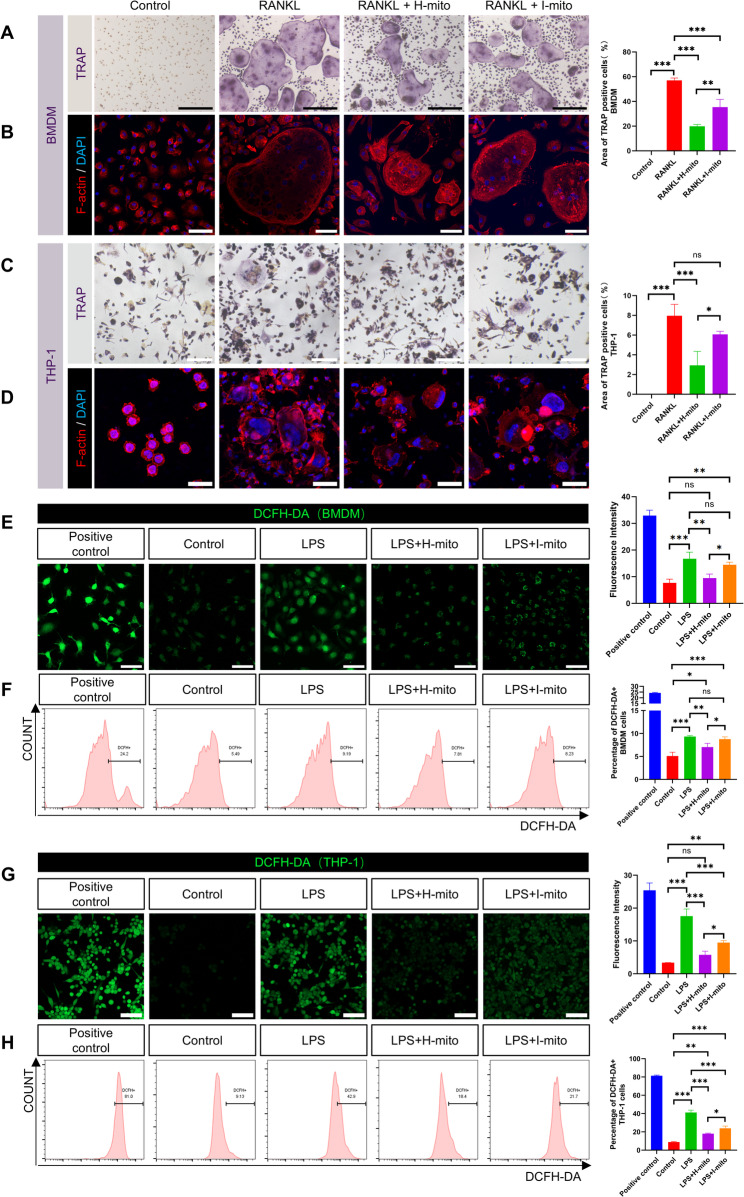
H-mito inhibits macrophage osteoclastogenesis and reduces ROS levels more potently. **A** TRAP staining results in BMDMs transplanted with isolated H-mito and I-mito (scale bar: 400 μm). **B** F-actin staining detection in BMDMs transplanted with isolated H-mito and I-mito by confocal microscopy (scale bar: 50 μm). **C** TRAP staining results in THP-1 cells transplanted with isolated H-mito and I-mito (scale bar: 200 μm). **D** F-actin staining detection in THP-1 cells transplanted with isolated H-mito and I-mito by confocal microscopy (scale bar: 50 μm). **E** ROS levels labeled by DCFH-DA (green fluorescent probe) were detected by confocal microscopy in BMDMs (scale bar: 50 μm). **F** Flow cytometry of the proportions of DCFH-DA-positive cells in BMDMs. **G** ROS levels labeled by DCFH-DA (green fluorescent probe) were detected by confocal microscopy in THP-1 cells (scale bar: 100 μm). **H** Flow cytometry of the proportions of DCFH-DA-positive cells in THP-1 cells. Cells treated with H₂O₂ served as the Positive control. ns: no significance, *P < 0.05, ** P < 0.01, *** P < 0.001

Since the mitochondrial transfer from PDLSCs to macrophages is impaired under inflammatory conditions, we hypothesized that exogenous mitochondrial supplementation could restore macrophage function. To test this hypothesis, osteoclast differentiation was induced in BMDMs and THP-1 cells using RANKL, in the presence of H-mito or I-mito. Both cell types successfully differentiated into osteoclasts, as confirmed by F-actin ring formation. Importantly, H-mito significantly suppressed osteoclastogenesis, whereas I-mito showed a weaker inhibitory effect (Fig. 2A and D). The results suggested that mitochondria from healthy PDLSCs inhibit macrophage-derived osteoclast differentiation more effectively than mitochondria from inflammatory PDLSCs.

Excessive accumulation of ROS is a hallmark of periodontitis, and current treatments aim to control the resulting inflammatory damage. To assess how H-mito and I-mito regulate ROS levels in macrophages under inflammatory conditions, we used LPS stimulation to mimic oxidative stress (Figure S2). Total intracellular ROS levels were evaluated using DCFH-DA staining and flow cytometry. LPS significantly increased ROS levels in BMDM and THP-1 cells while H-mito notably reduced the elevation. I-mito showed a suppressive effect, though to a lesser extent (Fig. 2E and H). MitoSOX Red was used to assess the mitochondrial reactive oxygen species (mtROS) production. Flow cytometry confirmed that H-mito effectively suppressed mtROS in both BMDM and THP-1 cells, whereas I-mito exhibited weaker antioxidant effects (Figure S3). The findings collectively indicated that H-mito confers superior redox protection in an inflammatory environment.

**Fig. 3:**
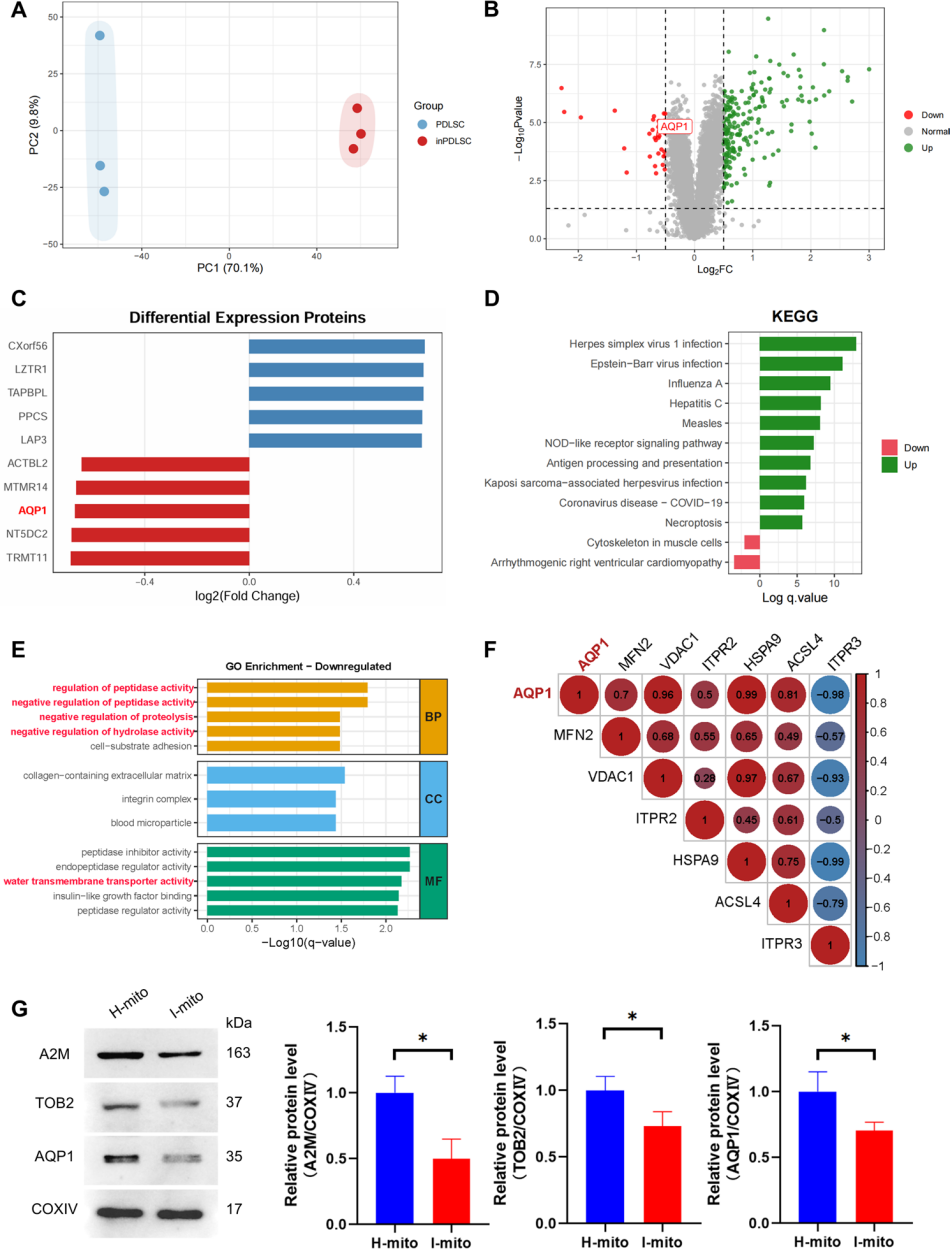
Quantitative proteomic sequencing reveals differential protein expression between H-mito and I-mito. **A** Principal component analysis for H-mito and I -mito.**B** Volcano plot showing non-regulated proteins (gray) and significantly up (green) and downregulated (red) proteins between H-mito and I -mito. The X axis represents log_2_-transformed fold change values. Y axis shows the−log_10_ p-value adjusted for multiple comparisons. **C** Differential expression of selected proteins in H-mito and I -mito.**D** KEGG enrichment analysis showing significantly up (green) and downregulated (red) pathways between H-mito and I -mito.**E** Gene Ontology enrichment analysis of down-regulated proteins. The pathways highlighted in red involve the activity of AQP1 protein. **F** Correlation analysis of hub genes.**G** Western blot analysis of A2M, TOB2 and AQP1 protein expression levels in H-mito and I-mito. ns: no significance, *P < 0.05

To investigate the intrinsic differences between H-mito and I-mito, we performed mitochondrial proteomic profiling. Among the differentially expressed proteins, A2M, TOB2 and AQP1 were notably upregulated in H-mito (Fig. 3). These results were further validated by Western blotting (Fig. 3G). Notably, among the three proteins, AQP1 showed the most well-established association with immune regulation, particularly in macrophage biology, according to previous literature [40]. AQP1 has been implicated in modulating macrophage polarization, migration, and inflammatory responses under various pathological conditions. Moreover, AQP1 is associated with molecules related to mitochondria and the endoplasmic reticulum (Fig. 3F).This strong literature foundation, together with its marked enrichment in H-mito, led us to prioritize AQP1 for further functional investigation in the context of periodontitis. Given the recent promising advances in targeting nuclear genes encoding mitochondrial proteins in disease research, enhancing nuclear AQP1 expression may serve as an effective strategy to regulate mitochondrial protein levels [41]. We established ovAQP1-PDLSCs via lentiviral transduction and successfully isolated ovAQP1-mito with preserved structural integrity and bioactivity (Figure S4-6).

## ovAQP1-mito transplantation inhibits macrophage osteoclastogenesis and M1 polarization in inflammatory conditions

**Fig. 4:**
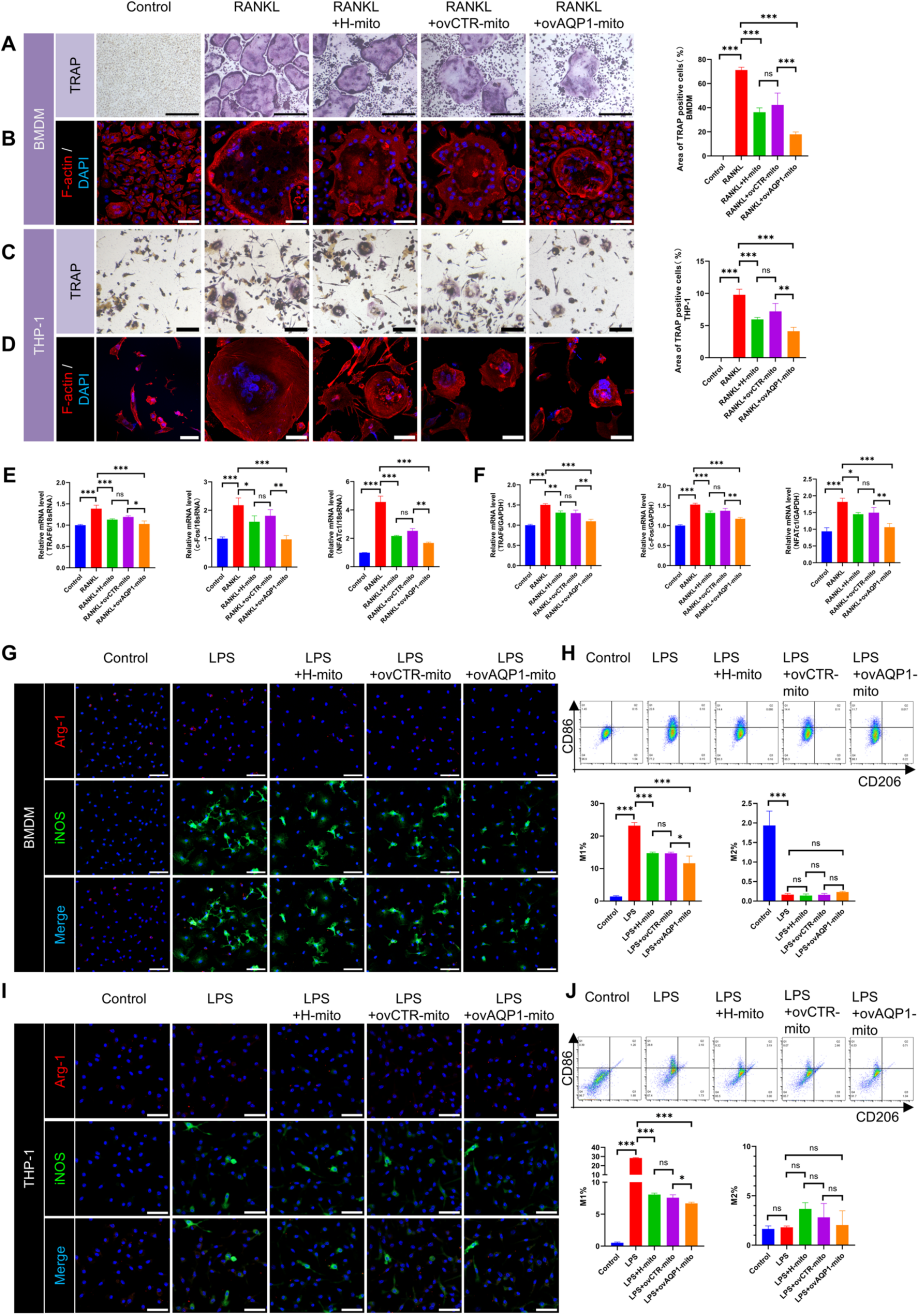
OvAQP1-mito transplantation inhibits macrophage osteoclastogenesis and macrophage M1 polarization in inflammatory conditions. **A** TRAP staining results in BMDMs transplanted with isolated H-mito, ovCTR-mito, and ovAQP1-mito (scale bar: 400 μm).**B** F-actin staining detection in BMDMs transplanted with isolated H-mito, ovCTR-mito, and ovAQP1-mito by confocal microscopy (scale bar: 50 μm). **C** TRAP staining results in THP-1 cells transplanted with isolated H-mito, ovCTR-mito, and ovAQP1-mito (scale bar: 200 μm).**D** F-actin staining detection in THP-1 cells transplanted with isolated H-mito, ovCTR-mito, and ovAQP1-mito by confocal microscopy (scale bar: 50 μm). **E** Relative mRNA expression of osteoclast‑differentiation–associated genes in BMDMs.**F** Relative mRNA expression of osteoclast‑differentiation–associated genes in THP-1 cells. **G** Immunofluorescence of macrophages polarization (left, scale bar: 50 μm). **H** Flow cytometry analysis of macrophages polarization and quantitative results (right). **I** Immunofluorescence of macrophages polarization (left, scale bar: 40 μm).**J** Flow cytometry of macrophages polarization and quantitative results (right). ns: no significance, *P < 0.05, ** P < 0.01, *** P < 0.001

To elucidate the functional role of AQP1 in mitochondria, we systematically evaluated the effects of three mitochondrial sources including H-mito, ovCTR-mito and ovAQP1-mito on osteoclast differentiation and macrophage polarization. Using TRAP staining and F-actin staining analysis, we assessed the effects of these mitochondria on BMDMs osteoclastogenesis. As expected, osteoclast induction remarkably promoted the formation of multinucleated TRAP-positive cells. Both H-mito and ovCTR-mito reduced the number and area of TRAP-positive cells, with ovAQP1-mito exhibiting the most potent inhibitory effect (Fig. 4A, C). Consistently, F-actin staining revealed that ovAQP1-mito strongly suppressed the formation of characteristic resorptive actin rings in mature osteoclasts, reinforcing its anti-osteoclastic function (Fig. 4B, D). RT-qPCR further demonstrated that ovAQP1-mito downregulated the key osteoclastogenic genes TRAF6, NFATc1 and c-Fos during differentiation (Fig. 4E, F).

Next, we evaluated the effects of different mitochondrial sources on macrophage polarization. Following LPS stimulation, BMDMs exhibited a remarkable increase in the number of M1-type macrophages, as detected by immunofluorescence. All three mitochondrial treatments, H-mito, ovCTR-mito and ovAQP1-mito, attenuated M1 polarization to varying degrees, with ovAQP1-mito demonstrating the most substantial inhibitory effect (Fig. 4G). Flow cytometric analysis corroborated these findings, revealing that ovAQP1-mito significantly reduced the proportion of the M1-type macrophages, outperforming the other two mitochondrial preparations (Fig. 4H). Similarly, in LPS-treated THP-1 cells, ovAQP1-mito showed the strongest suppression of M1 macrophage polarization (Fig. 4I, J). Notably, under the current experimental conditions, none of the mitochondrial treatments influenced M2 polarization significantly. The results suggested that ovAQP1-mito primarily exerts immunomodulatory effects by inhibiting pro-inflammatory M1 polarization rather than promoting M2 phenotypic switching.

Taken together, the results provided compelling evidence that ovAQP1-mito possess enhanced immunomodulatory properties. They not only effectively inhibited the osteoclastic differentiation of macrophages but also significantly alleviated LPS-induced M1 polarization. The findings underscored the critical role of AQP1 in maintaining mitochondrial function and regulating inflammation, and highlighted its therapeutic potential in controlling pathological bone resorption and immune activation.

### ovAQP1-mito transplantation alleviates periodontal bone resorption in a mouse model of experimental periodontitis

To assess the in-vivo efficacy of mitochondrial transplantation in periodontitis, a ligature-induced mouse model of periodontitis was established using 6-week-old C57BL/6 mice. The animals were randomized into six groups, namely NC (healthy control), PD (untreated periodontitis), PD + PBS (PBS injection) and three mitochondrial treatment groups that received local injections of H-mito, ovCTR-mito or ovAQP1-mito. After four weeks, the major organs and peripheral blood samples were collected. H&E staining revealed intact tissue architecture without inflammatory infiltration (Figure S7A), and blood analysis showed no significant change in hematological parameters (Figure S7B), indicating favorable biosafety and systemic compatibility.

**Fig. 5:**
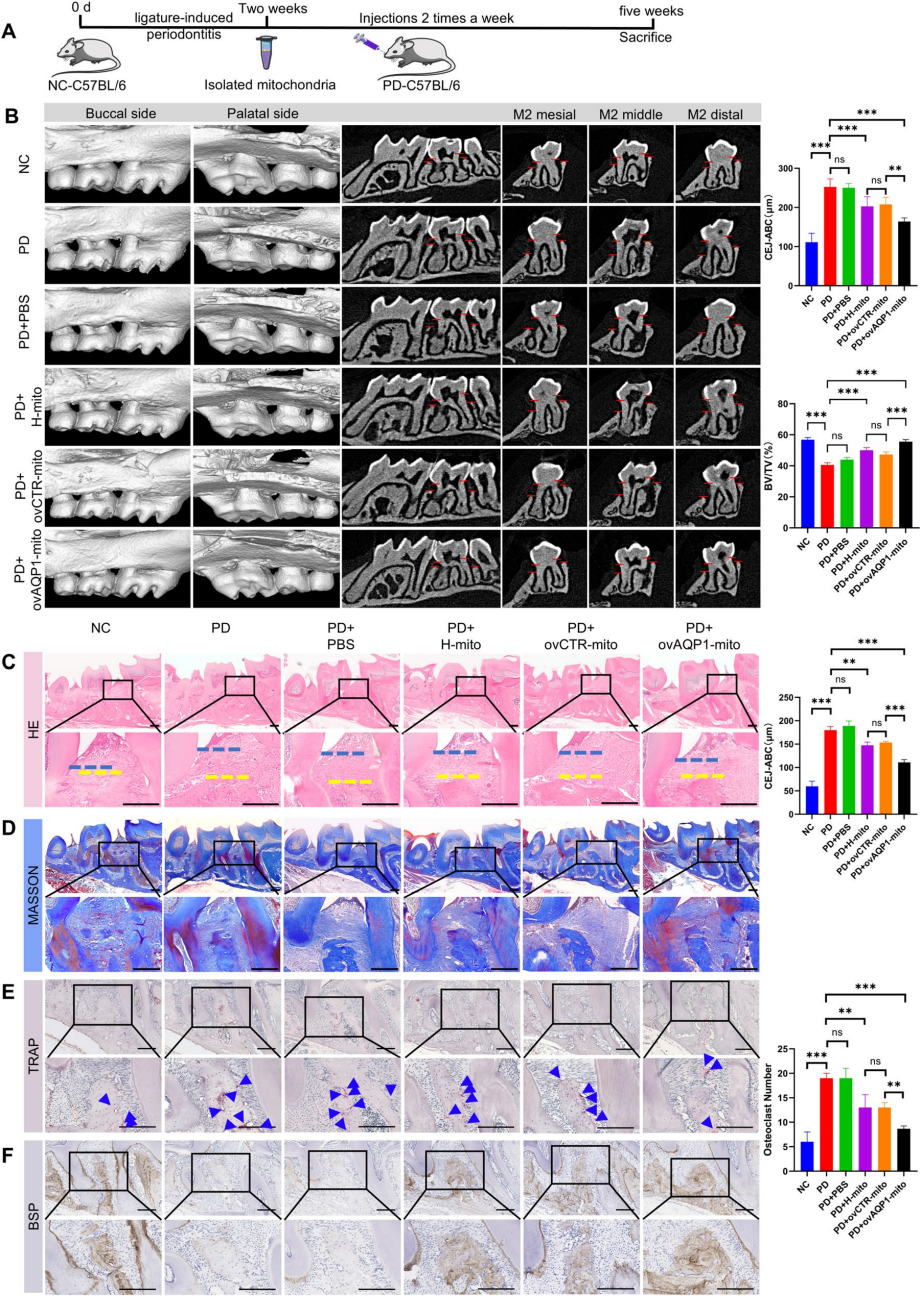
OvAQP1-mito transplantation alleviates periodontal bone resorption in a mouse model of experimental periodontitis. **A** Schematic diagram of the experimental model.**B** Micro-CT assessment of alveolar bone volume in mice from different groups. The red lines indicate the CEJ or ABC (n = 6 per group). Quantification of the CEJ–ABC distance and BV/TV is shown.**C** H&E staining of periodontal tissues from each group (scale bar: 200 μm; Blue and yellow dashed lines denote the CEJ–ABC distance. Quantification of the CEJ–ABC distance is shown.**D** Masson staining of periodontal tissues (scale bar: 200 μm).**E** TRAP staining of periodontal tissues (scale bar: 200 μm; Blue arrows indicate TRAP-positive osteoclasts). Quantification of osteoclast number is shown.**F** BSP staining of periodontal tissues (scale bar: 200 μm). For histological analyses (B–E), representative images were obtained from 3 mice per group. ns: not significant; *P < 0.05, ** P < 0.01, *** P < 0.001

Micro-CT results showed that the CEJ-ABC distances increased in the PD group, indicating bone loss, which was partially alleviated by H-mito and ovCTR-mito, ovAQP1-mito showing the strongest protective effect (Fig. 5B). Histological analysis using H&E and Masson staining revealed reduced bone height and disorganized collagen in the PD group, whereas ovAQP1-mito treatment substantially restored alveolar bone height and normalized collagen architecture (Fig. 5C, D). TRAP staining demonstrated elevated osteoclast numbers in PD group, which were significantly reduced by mitochondrial treatment, especially in ovAQP1-mito group (Fig. 5E). Immunohistochemistry showed decreased BSP expression in the PD group. All mitochondrial treatments partially restored BSP levels, with ovAQP1-mito producing the most pronounced recovery (Fig. 5F). Collectively, ovAQP1-mito transplantation suppressed osteoclast activity and promoted osteogenesis, helping restore periodontal bone homeostasis.

## ovAQP1-mito restores MERCs in macrophages via ATF4/SigmaR1 regulation

**Fig. 6:**
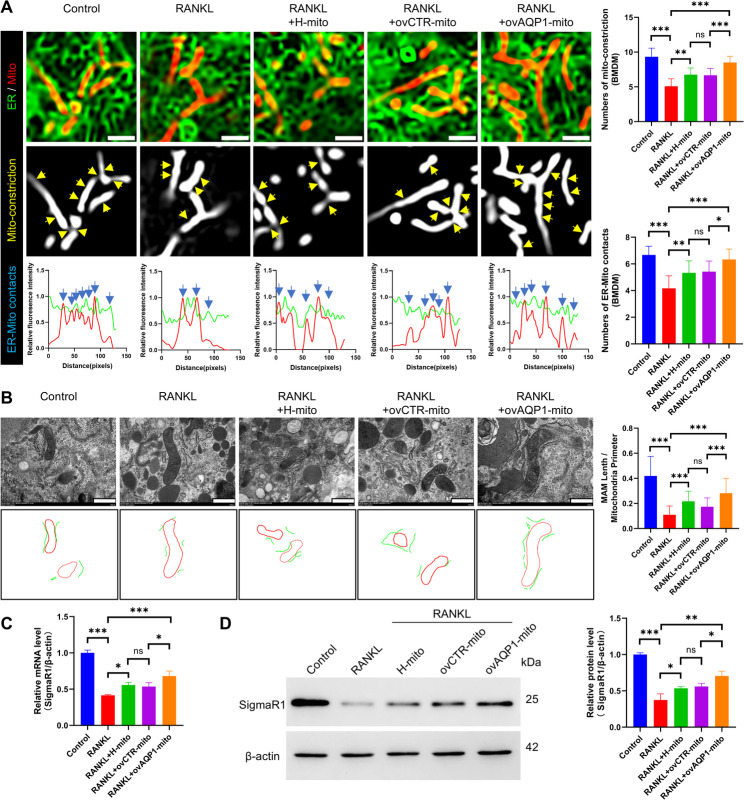
OvAQP1-mito transplantation modulates mitochondria-ER contacts during osteoclast differentiation in BMDMs. **A** Representative structured illumination microscopy images of mitochondria-ER contacts in BMDMs (scale bar: 1 μm; the endoplasmic reticulum was labeled with ER-Tracker Green and mitochondria were labeled with Mitotracker Deep Red. Each yellow arrow indicates a mito-constriction, each blue arrow indicates a mitochondria-ER contact). Mitochondrial constriction and mitochondria-ER contacts were enumerated in the region of interest (ROI) by ImageJ (n = 12 microscopic fields each).**B** Mitochondria-ER contacts in BMDMs under transmission electron microscopy (scale bar: 600 nm). Insets are mitochondrial membrane (red) and ER membrane (green). The MAM length / mitochondrion perimeter (46 mitochondria were analyzed) were further analyzed by ImageJ software.**C** Relative mRNA expression of SigmaR1 in BMDMs.**D** Western blot analysis of SigmaR1 protein expression levels and quantitative analysis in BMDMs. ns: no significance, *P< 0.05, ** P < 0.01, *** P < 0.001

MERCs serve as essential structural platforms for Ca²⁺ signaling transients that trigger osteoclastogenesis in macrophages [42, 43]. To determine whether the inhibitory effect of ovAQP1-mito on osteoclast differentiation is related to alterations in MERCs integrity, we visualized mitochondria-ER contacts in macrophages using structured illumination microscope. Results demonstrated that RANKL stimulation disrupted the mitochondria-ER contacts, indicating a compromised MERCs structure. In contrast, ovAQP1-mito treatment increased mitochondria-ER contacts and mitochondrial constriction (Fig. 6A). Mitochondria-associated ER membranes (MAMs), which represent the interface between mitochondria and ER with an approximate spacing of 10–50 nm, were further assessed by transmission electron microscopy to evaluate the effect of ovAQP1-mito on MAMs dynamics. The proportion of mitochondria located within 50 nm of the ER was significantly elevated after ovAQP1-mito treatment. Morphologically, mitochondria in the ovAQP1-mito group were frequently wrapped by ER, leading to a notable increase in MAM length compared to the RANKL group, indicating structural remodeling of MERCs (Fig. 6B). Notably, ovAQP1-mito treatment partially restored mitochondrial-ER contacts in RANKL-stimulated cells, suggesting a protective effect on MERC architecture (Fig. 6A, B).

SigmaR1, a chaperone protein that facilitates MERC formation, has been reported to negatively regulate osteoclastogenesis. Previous studies had shown that SigmaR1 knockdown reduces mitochondria-ER contacts [44]. Based on these observations, we hypothesized that ovAQP1-mito suppresses osteoclast differentiation by restoring RANKL-disrupted MERCs via SigmaR1 modulation. RT-qPCR and western blot analyses revealed a significant downregulation of SigmaR1 following RANKL treatment, whereas ovAQP1-mito intervention partially reversed the decrease (Fig. 6C, D).

**Fig. 7:**
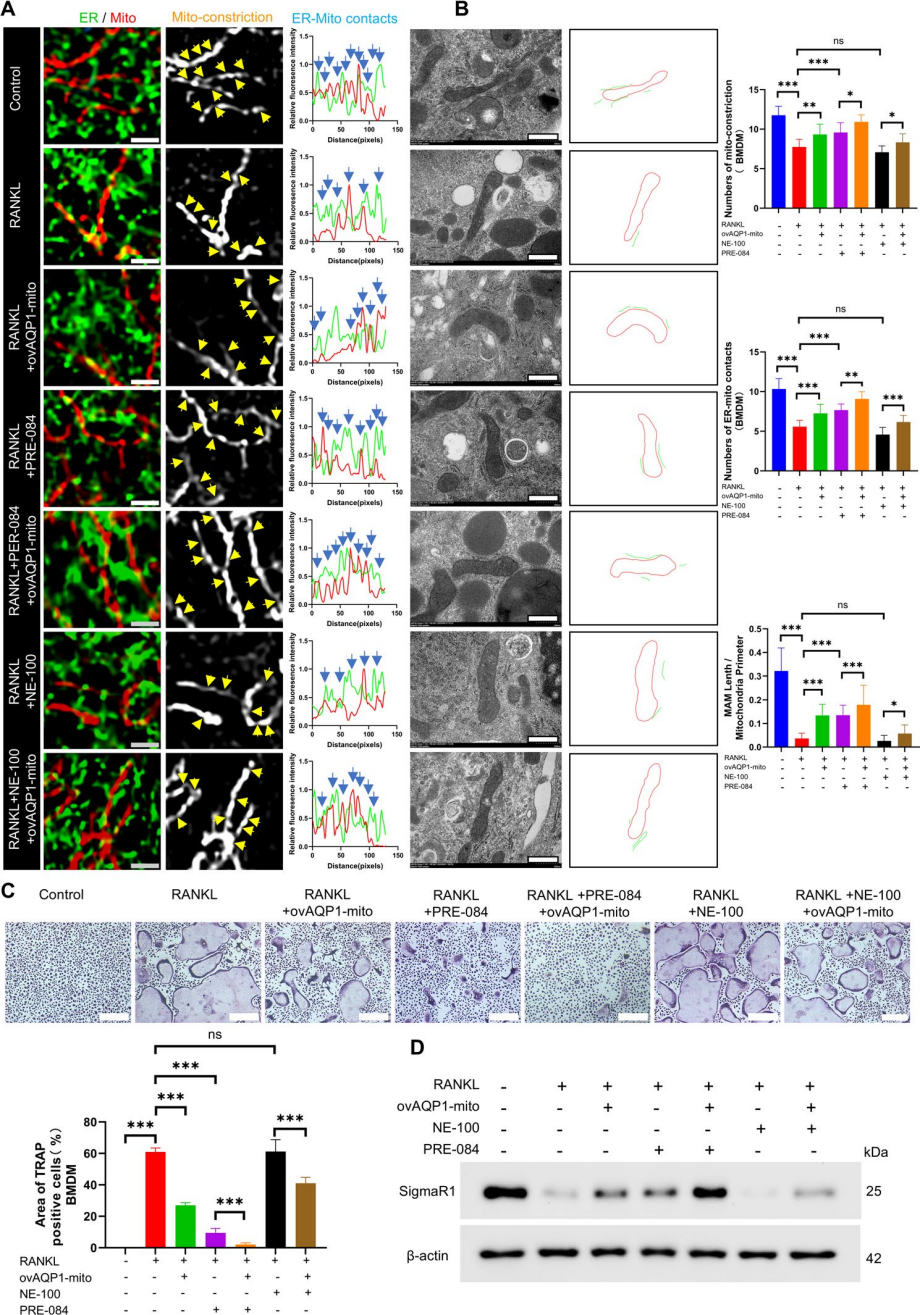
SigmaR1 serves as a key mediator in ovAQP1-mito–regulated MERCs. **A** Representative structured illumination microscopy images of mitochondria-ER contacts in BMDMs (scale bar: 0.5 μm; the endoplasmic reticulum was immunolabeled with anti-calnexin and a green fluorescent secondary antibody and mitochondria were labeled with Mitotracker Deep Red. Each yellow arrow indicates a mito-constriction, each blue arrow indicates a mitochondria-ER contact). Mitochondrial constriction and mitochondria-ER contacts were enumerated in the region of interest (ROI) by ImageJ (n = 12 microscopic fields each).**B** Mitochondria-ER contacts in BMDMs under transmission electron microscopy (scale bar: 600 nm). Insets are mitochondrial membrane (red) and ER membrane (green). The MAM length / mitochondrion perimeter (50 mitochondria were analyzed) were further analyzed by ImageJ software.**C** TRAP staining of BMDMs from different experimental groups (scale bar: 300 μm).**D** Western blot analysis of SigmaR1 protein expression levels in BMDMs. ns: no significance, *P< 0.05, ** P < 0.01, *** P < 0.001

To further elucidate the functional role of SigmaR1 in ovAQP1-mito-mediated inhibition of osteoclastogenesis, pharmacological modulation was performed using the SigmaR1 agonist PRE-084 and the antagonist NE-100. To directly assess whether pharmacological modulation of SigmaR1 alters mitochondria–ER contacts, we first employed structured illumination microscopy and transmission electron microscopy. Fluorescence imaging revealed that PRE-084 treatment markedly enhanced the colocalization between mitochondria and ER, resembling the effect observed in the ovAQP1-mito group, whereas NE-100 administration disrupted these contacts (Fig. 7A). Consistently, TEM analysis showed that PRE-084 increased the proportion of mitochondria located within 50 nm of the ER and significantly extended MAM length compared with the RANKL group. In contrast, NE-100 treatment diminished ER wrapping around mitochondria and reduced MAM length, while ovAQP1-mito co-treatment partially restored ER–mitochondria connectivity (Fig. 7B). Functionally, TRAP staining revealed that both PRE-084 and ovAQP1-mito significantly inhibited osteoclast formation. Conversely, co-treatment with ovAQP1-mito and NE-100 abolished the anti-osteoclastogenic effect, underscoring the essential role of SigmaR1 in this regulatory pathway (Fig. 7C). In parallel, Western blot analysis of BMDMs collected after pharmacological modulation demonstrated that ovAQP1-mito synergized with PRE-084 to upregulate SigmaR1 expression, while attenuating the downregulation induced by NE-100 (Fig. 7D). Together with the TRAP staining results, these findings indicate that ovAQP1-mito regulates SigmaR1 expression in BMDMs, thereby modulating osteoclast differentiation.

**Fig. 8:**
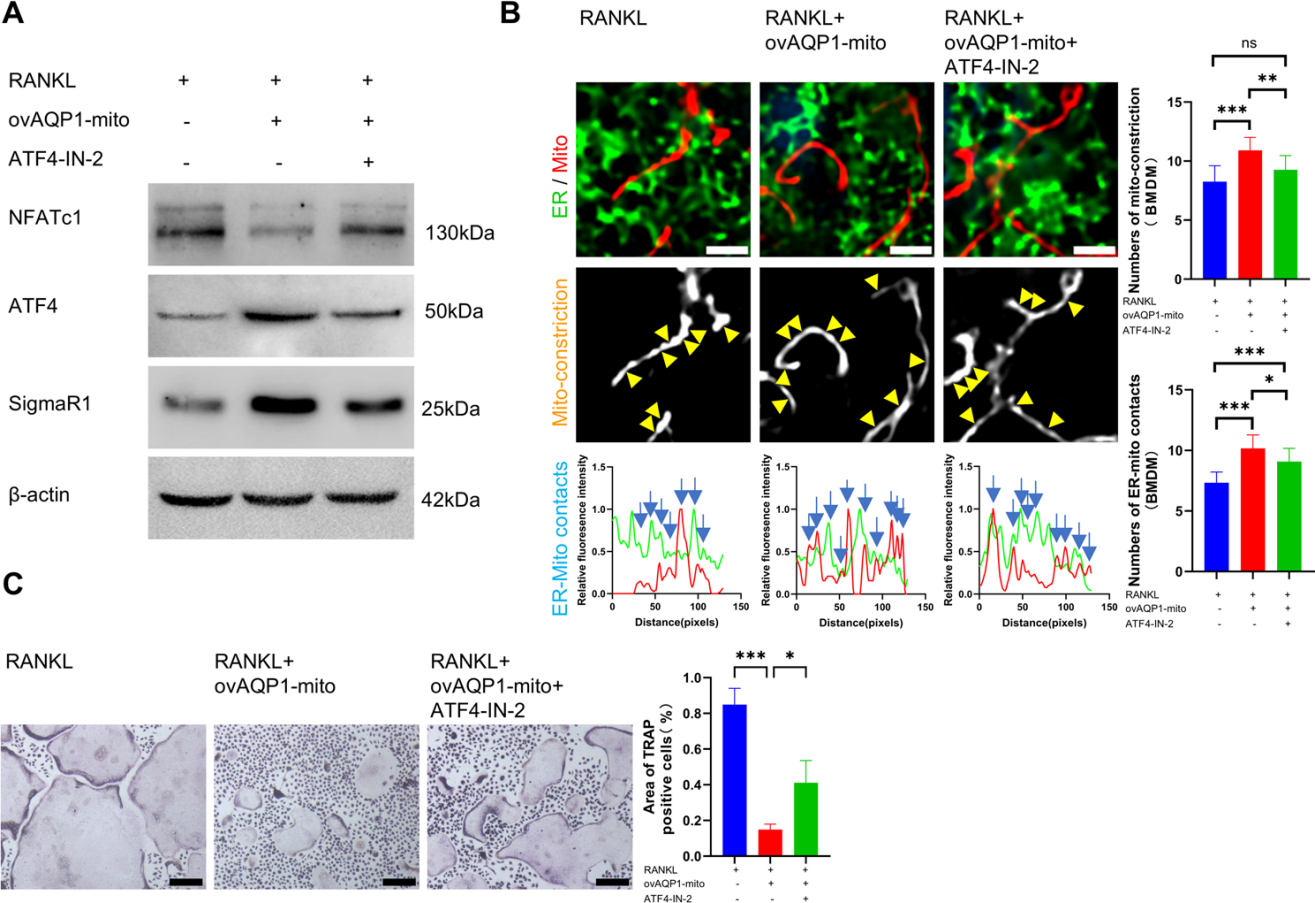
OvAQP1-mito modulates macrophage osteoclastogenesis through the ATF4/SigmaR1/ER-mitochondria contact axis. **A** Western blot analysis of protein expression levels in BMDMs.**B** Representative structured illumination microscopy(SIM) images of mitochondria-ER contacts in BMDMs (scale bar: 0.5 μm; the endoplasmic reticulum was immunolabeled with anti-calnexin and a green fluorescent secondary antibody and mitochondria were labeled with Mitotracker Deep Red. Each yellow arrow indicates a mito-constriction, each blue arrow indicates a mitochondria-ER contact). Mitochondrial constriction and mitochondria-ER contacts were enumerated in the region of interest (ROI) by ImageJ (n = 12 microscopic fields each).**C** TRAP staining of BMDMs from different experimental groups (scale bar: 200 μm). ns: no significance, *P < 0.05, *** P < 0.001

ATF4 plays a dual role in bone metabolism, promoting osteogenesis while inhibiting osteoclastogenesis [45], and has been identified as a key upstream transcription factor regulating SigmaR1 [46, 47]. To determine whether the ATF4/SigmaR1 axis mediates ovAQP1-mito–induced SigmaR1 upregulation, we examined ATF4 expression in RANKL-stimulated BMDMs. Western blot analysis showed that ovAQP1-mito markedly increased ATF4 and SigmaR1 protein levels in RANKL-stimulated BMDMs. Pharmacological inhibition of ATF4 using ATF4-IN-2 abolished ovAQP1-mito–induced SigmaR1 upregulation, indicating that ATF4 is required for ovAQP1-mito–mediated SigmaR1 induction in BMDMs (Fig. 8A). Consistently, SIM imaging demonstrated that ATF4 inhibition largely impaired the ovAQP1-mito–mediated restoration of RANKL-induced mitochondria–ER contact (MERC) disruption (Fig. 8B). Functionally, TRAP staining further confirmed that ATF4 inhibition reversed the suppressive effect of ovAQP1-mito on osteoclast formation in BMDMs (Fig. 8C).

Given the limited efficiency of siRNA transfection in primary BMDMs, we employed RANKL-stimulated human THP-1–derived macrophages as a complementary model to further interrogate the ATF4/SigmaR1 axis using both pharmacological and genetic approaches. Western blot analysis revealed that ovAQP1-mito similarly increased ATF4 and SigmaR1 protein levels in THP-1 cells. Both ATF4 inhibition with ATF4-IN-2 and siRNA-mediated knockdown of ATF4 markedly attenuated ovAQP1-mito–induced SigmaR1 upregulation, confirming the requirement of ATF4 for SigmaR1 induction in human cells (Figure S8A, B). Moreover, SIM imaging showed that ovAQP1-mito significantly restored MERC integrity disrupted by RANKL stimulation in THP-1 cells, whereas both pharmacological inhibition and genetic silencing of ATF4 largely abolished this effect (Figure S8C). Functionally, TRAP staining demonstrated that suppression of osteoclast differentiation by ovAQP1-mito was significantly reversed upon ATF4 inhibition or knockdown in THP-1 cells (Figure S8D).

## Discussion

Our study demonstrated that mitochondrial transplantation from PDLSCs to macrophages is impaired under inflammation. A comparative analysis revealed that H-mito protected mitochondrial function and suppressed osteoclast differentiation more effectively than I-mito. Proteomic profiling identified AQP1 as a key protein enriched in H-mito. Functional experiments with ovAQP1-mito revealed enhanced mitochondria-ER contacts, and significant inhibition of osteoclastogenesis in vitro and alveolar bone loss in vivo. The findings suggested ovAQP1-mito as a novel therapeutic tool for modulating bone homeostasis in periodontitis.

Periodontitis activates the TLR2/4 pathway via microbial components, such as EVs, LPS, and PGDHC, inducing RANKL expression and promoting osteoclastogenesis and alveolar bone resorption [48–50]. Inflammatory conditions also impair mitochondrial function in PDLSCs and macrophages, characterized by metabolic disruption, mtDNA release, oxidative stress, mitophagy defects, and dysregulated Mfn1/2 and PINK1 expression, thereby exacerbating inflammation and impeding repair [51–53], with macrophages particularly affected and prone to M1 polarization. In our study, ligature-induced periodontitis in mice led to increased osteoclast and alveolar bone losses. LPS-treated PDLSCs exhibited reduced proliferation and osteogenic capacity; ultrastructurally, mitochondrial rupture and crista loss were evident. Inflammatory macrophages showed elevated ROS and mtROS levels and M1 polarization. Although intercellular mitochondrial transfer can support damaged cells, excessive mitochondrial transfer in pathological states, such as osteoporosis, disrupts MSC metabolism and inhibits osteogenesis [54]. While macrophages take up mitochondria efficiently under healthy conditions, the capacity declines in diseases like obesity [55, 56]. In this study, we observed that inflammation impaired mitochondrial transfer from PDLSCs to macrophages, which suggested that exogenous transplantation of healthy mitochondria could restore macrophage function, suppress inflammation, and reduce bone loss, thereby offering a potential therapeutic strategy.

Exogenous mitochondrial transplantation, particularly using mitochondria derived from MSCs, can restore mitochondrial function in recipient cells in the context of various diseases. In macrophages, MSC-derived mitochondria elevate mtDNA content, mitochondrial membrane potential (MMP), and oxidative phosphorylation (OXPHOS) activity, thereby enhancing energy metabolism and ATP production [57, 58]. Additionally, donor-derived antioxidant proteins, such as SOD2 and SIRT2, alleviate oxidative stress, whereas MSC-associated microRNAs suppress TLR signaling and promote anti-inflammatory polarization [59–61]. Previous studies had reported that mitochondria from bone marrow MSCs can enhance phagocytosis and reduce inflammation in alveolar macrophages, supporting their therapeutic potential in bone loss–related inflammatory diseases [57, 58]. In our study, the transplantation of H-mito into macrophages exposed to inflammatory and osteoclastogenic conditions significantly reduced ROS and mtROS levels and inhibited osteoclastic differentiation. In contrast, I-mito exhibited limited efficacy, suggesting that mitochondrial quality critically determines therapeutic outcomes. Proteomic analysis revealed that AQP1 is significantly upregulated in H-mito than in I-mito. This highlighted AQP1 as a potential regulatory factor in mitochondrial function and macrophage fate, meriting further mechanistic investigations.

We used nuclear gene editing to construct ovAQP1-mito and assessed their effects on macrophage osteoclastogenesis. ovAQP1-mito significantly inhibited osteoclast differentiation while ovCTR-mito showed only mild effects. Calcium (Ca²⁺) is central to osteoclast regulation. Mitochondrial electron transport defects cause abrupt Ca²⁺ release into the cytosol, activating the Ca²⁺/calcineurin/NFATc1 pathway and promoting osteoclastogenesis [62]. Mitochondria, ER, and lysosomes store Ca²⁺, and mitochondria - ER interactions via MAMs facilitate Ca²⁺, lipid, and metabolite exchange, maintaining metabolic homeostasis [42, 63]. Accordingly, we hypothesized that Ca²⁺ signaling during osteoclastogenesis is involved in MAM remodeling. Indeed, during ovAQP1-mito intervention in BMDMs, osteoclast differentiation correlated with reduced ER-mito contacts, whereas ovAQP1-mito restored the contact, indicating a role in MAM regulation. In line with this, ROS transfer at peroxisome–mitochondria contact sites regulates mitochondrial redox status, which may offer a potential therapeutic strategy for periodontitis [5, 64].

Further evidence points to SigmaR1, an intracellular chaperone protein localized in the ER, MAMs and nuclear membrane, as a critical regulator of Ca²⁺ signaling, mitochondrial integrity and energy metabolism [65]. SigmaR1 directly promotes ER-mito contact, and its loss or knockdown significantly reduces the interactions [44, 66]. Therefore, we speculated that the restored ER-mito contact and the associated suppression of osteoclastogenesis observed with ovAQP1-mito could be mediated through SigmaR1 activation. Interestingly, SigmaR1 has also been implicated in bone homeostasis. Mice lacking SigmaR1 exhibit severe bone loss in models of ovariectomy-induced osteoporosis, whereas local overexpression of SigmaR1 alleviates osteoporotic phenotypes [67]. In our study, the use of SigmaR1 agonists and antagonists further confirmed that the inhibitory effect of ovAQP1-mito on osteoclast formation is dependent on SigmaR1 activity, supporting a mechanism by which ovAQP1-mito activates SigmaR1, restores the mitochondria-ER contact, and suppresses osteoclastogenesis.

Activating transcription factor 4 (ATF4) is a central transcription factor of the integrated stress response (ISR), classically activated by eIF2α phosphorylation–dependent selective translation [68, 69]. Beyond ER stress, mitochondrial dysfunction alone can induce ATF4 activation through mitochondrial retrograde signaling [70, 71], as changes in mitochondrial membrane potential, oxidative phosphorylation, and ROS or Ca²⁺ microenvironments enhance ATF4 translation independently of the canonical UPR [70, 72]. Here, ovAQP1-mito markedly upregulated ATF4 in RANKL-induced BMDMs, suggesting activation of an adaptive ATF4 response via modulation of mitochondrial functional states. Given the role of AQP1 in regulating water flux, osmotic balance, and Ca²⁺ dynamics, ovAQP1-mito may reshape RANKL-induced mitochondrial dysfunction to activate the eIF2α–ATF4 axis. Under stress conditions, ATF4 accumulates in the nucleus and enhances transcription through AARE/CRE elements [73]). Importantly, ATF4 directly binds the 5′-flanking region of the SigmaR1 (Sig-1R) gene and promotes its transcription, with ATF4 activation closely correlating with SigmaR1 upregulation [46, 74]. Consistently, ovAQP1-mito concomitantly increased ATF4 and SigmaR1 expression and restored RANKL-disrupted ER–mitochondria contacts. Collectively, ovAQP1-mito may suppress osteoclast differentiation by activating the ATF4–SigmaR1 transcriptional axis to reestablish ER–mitochondria coupling.

However, the mechanism of exogenous mitochondria in recipient cells remain unclear. Previous studies reported that exogenous mitochondria did not integrate into the endogenous mitochondrial pool, but triggered mitophagy after internalization. Functionally impaired mitochondria can still improve host adaptability and cytoprotective effect [38]. Here we showed that, at least in part, Instead, AQP1 may act as the key effector. Taken together, ovAQP1-mito is likely to significantly modulate MERCs/Ca²⁺ microdomains, ultimately suppressing osteoclast differentiation and M1 polarization.

## Conclusions

In summary, our findings illustrated the construction of engineered mitochondria overexpressing AQP1 and revealed a mechanistic paradigm in which exogenous ovAQP1-mito modulates macrophage osteoclastogenesis through activation of the ATF4/SigmaR1 axis and subsequent enhancement of mitochondria–ER contacts. The regulation contributes to the attenuation of alveolar bone loss during periodontitis. Collectively, AQP1-overexpressing mitochondria may be a promising therapeutic tool against periodontitis and other inflammatory disorder leading to bone loss. Therapeutic transplantation of engineered mitochondria could represent a promising approach for restoring skeletal homeostasis in the context of inflammation.

## Supplementary Information

Supplementary Material 1

## Data Availability

No datasets were generated or analysed during the current study.

## Abbreviations

MSCs: Mesenchymal stromal cells
PDLSCs: Periodontal ligament stromal cell
I-PDLSCs: Pdlscs from patients with periodontitis
ovCTR-PDLSCs: H-pdlscs transfected with the control plasmid
ovAQP1-PDLSCs: H-pdlscs overexpressing AQP1
AQP1: Aquaporin 1
TOB2: Transducer of ERBB2, 2
A2M: Alpha-2-macroglobulin
ovCTR-mito: Mitochondria derived from ovCTR-PDLSCs
ovAQP1-mito: Mitochondria derived from ovAQP1-PDLSCs
H-Mito: Mitochondria derived from H-PDLSCs
I-Mito: Mitochondria derived from I-PDLSCs
PINK1: PTEN induced putative kinase 1
BMDMs: Bone marrow-derived macrophages
THP-1: Human Acute Monocytic Leukemia Cells
M-CSF: Macrophage colony-stimulating factor
RANKL: Nuclear factor kappa-B ligand
PMA: Phorbol-12-myristate-13-acetate
ROS: Reactive oxygen species
Pg.LPS: Lipopolysaccharide from P. gingivalis
DCFH-DA: 2′,7′-dichlorofluorescindiacetate
TRAP: Tartrate resistant acid phosphatase
NFATc1: Nuclear factor of activated T cells 1
c-Fos: AP-1 transcription factor subunit
iNOS: Inducible nitric oxide sythase
Arg-1: Arginase 1
PD: Periodontitis
AST: Aspartate transaminase
CEJ-ABC: Cementum-enamel junction to alveolar bone crest distance
MERCs: Mitochondria–endoplasmic reticulum contacts
MAMs: Mitochondria-Associated Endoplasmic Reticulum Membrane
ER: Endoplasmic reticulum
SigmaR1: Sigma non-opioid intracellular receptor 1
EVs: Extracellular vesicles
PGDHC: Phosphoglycerol dihydroceramide
Mfn1/2: Mitofusin 1/2
PINK1: PTEN induced kinase 1
MMP: Mitochondrial membrane potential
OXPHOS: Oxidative phosphorylation
ATP: Adenosine 5’-triphosphate
SOD2: Manganese superoxide dismutase 2
SIRT2: NAD-dependent deacetylase sirtuin 2
H&E: Hematoxylin & eosin
BSP: Bone sialoprotein
