# Supplementary material for: Transplantation of AQP1-overexpressing mitochondria attenuates periodontitis-associated bone loss by regulating mitochondria-ER contacts of macrophages
Source: J Nanobiotechnology. 2026 May 28;24:726. doi: 10.1186/s12951-026-04084-z (PMC13449486; doi:10.1186/s12951-026-04084-z)
Supplement: Supplementary file 1 [file 12951_2026_4084_MOESM1_ESM.docx]

**Supplementary Information**

Additional file 1: Figure S1. Characterization of macrophage and mitochondrial transfer from PDLSCs to bone marrow cells. Figure S2. Establishment of periodontal inflammatory microenvironment using LPS stimulation. Figure S3. H-mito alleviates mitochondrial mtROS in inflammatory macrophages. Figure S4. Construction of AQP1-overexpressing PDLSCs and mitochondria and validation of mitochondrial Isolation. Figure S5. Characterization of H-PDLSCs, ovCTR-PDLSCs and ovAQP1-PDLSCs. Figure S6. Intercellular mitochondrial communication between AQP1-overexpressing PDLSCs and BMDM/THP-1 macrophages. Figure S7. Safety assessment of mitochondrial transplantation in mice with experimental periodontitis. Figure S8. OvAQP1-mito modulates macrophage osteoclastogenesis through the ATF4/SigmaR1/ER-mitochondria contact axis. Table S1. Primer sequence ^(10.8M, docx)^

**Additional file 1: Fig S1.** **Characterization of macrophage and mitochondrial transfer from PDLSCs to bone marrow cells.**

A. Morphology of BMDMs under light microscopy (left; scale bar: 10 μm). Flow cytometry detection of the expression proportions of surface markers CD11b and CD68 in BMDMs (right).

B. Morphology of THP-1 cells under light microscopy (left; scale bar: 100 μm). Flow cytometry detection of the expression proportions of surface markers CD14 and CD68 in THP-1 cells (right).

C. Confocal imaging of mitochondrial transfer from bone-lineage cells to myeloid cells (scale bar: 20 μm). MTR-Mito indicates mitochondria labeled with MitoTracker Deep Red.

D. Statistical analysis of the proportion of Mitotracker Red-positive cells in BMDMs, CD4^+^ T cells, CD19^+^ B cells and neutrophils.

**Additional File 1: Fig S2. Establishment of periodontal inflammatory microenvironment using LPS stimulation.**

A. Analysis of PDLSCs viability by the MTS assay at different concentrations of LPS.

B. Morphology of mitochondria in H-PDLSCs, I-PDLSCs and LPS-PDLSCs under transmission electron microscopy (left; scale bar: 600 nm) and statistical analysis of the proportion of healthy mitochondria in total mitochondria (right).

C. ALP staining of PDLSCs after osteogenic induction for 7 days (scale bar: 200 μm).

D. Alizarin red staining of PDLSCs after osteogenic induction for 21 days (scale bar: 400 μm).

ns: no significance, ** *P* < 0.01, *** *P* < 0.001.

**Additional File 1: Fig S3. H-mito alleviates mitochondrial** **mtROS in inflammatory macrophages.**

A. Flow cytometry detection of the proportions of MitoSOX Red-positive cells in BMDMs (left) and corresponding statistical analysis (right).

B. Flow cytometry detection of the proportions of MitoSOX Red-positive cells in THP-1 cells (left) and corresponding statistical analysis (right).

ns: no significance,** *P* < 0.01, *** *P* < 0.001.

**Additional file 1:** **Fig S4. Construction of AQP1-overexpressing PDLSCs and mitochondria and validation of mitochondrial Isolation.**

A. Western blot analysis of AQP1 protein expression levels and quantitative analysis in PDLSCs.

B. RT-qPCR analysis of AQP1 mRNA expression levels in PDLSCs.

C. Western blot analysis of AQP1 protein expression levels and quantitative analysis in isolated mitochondria.

D. Morphology of isolated mitochondria under transmission electron microscopy (scale bar: 300 nm).

E. Mitochondria isolated from PDLSCs were stained with Mitotracker Red and visualized by fluorescence microscopy to assess mitochondrial viability (scale bar: 50 μm).

F. Size distribution (left) and membrane potential levels (right) of isolated mitochondria by Zetaview.

ns: no significance, ***P* < 0.01, *** *P* < 0.001.

**Additional file 1: Fig S5. Characterization of H-PDLSCs, ovCTR-PDLSCs and ovAQP1-PDLSCs.**

A. Crystal violet staining was used to assess the colony-forming ability of H-PDLSCs, ovCTR-PDLSCs and ovAQP1-PDLSCs (scale bar: 400 μm).

B, C, D. Alizarin Red staining (B, scale bar: 200 μm), Oil Red O staining (C, scale bar: 60 μm) and Alcian Blue staining (D, scale bar: 100 μm) were used to assess osteogenic, adipogenic, and chondrogenic differentiation capacities of H-PDLSCs, ovCTR-PDLSCs and ovAQP1-PDLSCs, respectively.

E. Immunofluorescence staining was performed to identify the tissue origin of PDLSCs. PDLSCs expressed the mesenchymal marker vimentin (left) but did not express the epithelial marker cytokeratin 18 (right) (scale bar: 100 μm).

F. Flow cytometry was used to characterize the surface markers of PDLSCs.

**Additional file 1: Fig S6. Intercellular mitochondrial communication between AQP1-overexpressing PDLSCs and BMDM/THP-1 macrophages.**

A. Confocal imaging of mitochondrial communication between BMDMs and H-PDLSCs, ovCTR-PDLSCs and ovAQP1-PDLSCs (scale bar: 25 μm).

B. Confocal imaging of the uptake of isolated H-mito, ovCTR-mito and ovAQP1-mito by BMDMs (scale bar: 15 μm).

C. Confocal imaging of mitochondrial communication between THP-1 cells and H-PDLSCs, ovCTR-PDLSCs and ovAQP1-PDLSCs (scale bar: 20 μm);

D. Confocal imaging of the uptake of isolated H-mito, ovCTR-mito and ovAQP1-mito by THP-1 cells (scale bar: 15 μm).

**Additional file 1: Fig S7. Safety assessment of mitochondrial transplantation in mice with experimental periodontitis.**

A. H&E staining of paraffin sections of the heart, liver, spleen, lungs, kidney and colon of mice observed under light microscopy (scale bar: 200 μm);

B. Biochemical indicators and complete blood count tests of mouse blood. The red dashed line denotes the boundary of the normal value range.

**Additional file 1: Fig S8. OvAQP1-mito modulates macrophage osteoclastogenesis through the ATF4/SigmaR1/ER-mitochondria contact axis.**

A. Western blot analysis of ATF4 protein expression levels in THP-1.

B. Western blot analysis of protein expression levels in THP-1.

C. Representative structured illumination microscopy(SIM) images of mitochondria-ER contacts in THP-1(scale bar: 0.5 μm; the endoplasmic reticulum was immunolabeled with anti-calnexin and a green fluorescent secondary antibody and mitochondria were labeled with Mitotracker Deep Red. Each yellow arrow indicates a mito-constriction, each blue arrow indicates a mitochondria-ER contact). Mitochondrial constriction and mitochondria-ER contacts were enumerated in the region of interest (ROI) by ImageJ (n = 12 microscopic fields each).

D. TRAP staining of THP-1 from different experimental groups (scale bar: 200 μm).

ns: no significance, **P* < 0.05, ** *P* < 0.01, *** *P* < 0.001.

**Additional file 1: Table S1. Primer sequence**

|  | Primer |  |
| --- | --- | --- |
| Mouse | 18sRNA | Forward（5’-3’）:GTAACCCGTTGAACCCCATT  Reverse（5’-3’）: CCATCCAATCGGTAGTAGCG |
|  | | |
|  | TRAF6 | Forward（5’-3’）:TACGATGTGGAGTTTGACCCA  Reverse（5’-3’）: CACTGCTTCCCGTAAAGCCAT |
|  | | |
|  | c-Fos | Forward（5’-3’）:CGGGTTTCAACGCCGACTA  Reverse（5’-3’）:TGGCACTAGAGACGGACAGAT |
|  | | |
|  | NFATc1 | Forward（5’-3’）:GGAGAGTCCGAGAATCGAGAT  Reverse（5’-3’）: TTGCAGCTAGGAAGTACGTCT |
|  | | |
|  | β-actin | Forward（5’-3’）: CATGTACGTTGCTATCCAGGC  Reverse（5’-3’）: CTCCTTAATGTCACGCACGAT |
|  | | |
|  | SigmaR1 | Forward（5’-3’）:GACAGTATGCGGGGCTGGACCA  Reverse（5’-3’）: ACAACTGTCTCTCCTGGGTAGAAGA |
|  | | |
| Human | AQP1 | Forward（5’-3’）:TATGCGTGCTGGCTACTACC  Reverse（5’-3’）: CAGGGTTAATCCCACAGCCA |
|  | | |
|  | GAPDH | Forward（5’-3’）:GGAGCGAGATCCCTCCAAAAT  Reverse（5’-3’）: GGCTGTTGTCATACTTCTCATGG |
|  | | |
|  | TRAF6 | Forward（5’-3’）:TTTGCTCTTATGGATTGTCCCC  Reverse（5’-3’）: CATTGATGCAGCACAGTTGTC |
|  | | |
|  | c-Fos | Forward（5’-3’）:CCGGGGATAGCCTCTCTTACT  Reverse（5’-3’）: CCAGGTCCGTGCAGAAGTC |
|  | | |
|  | NFATc1 | Forward（5’-3’）:CACCGCATCACAGGGAAGAC  Reverse（5’-3’）: GCACAGTCAATGACGGCTC |
